# Comprehensive gene expression analysis of the *NAC* gene family under normal growth conditions, hormone treatment, and drought stress conditions in rice using near-isogenic lines (NILs) generated from crossing Aday Selection (drought tolerant) and IR64

**DOI:** 10.1007/s00438-012-0686-8

**Published:** 2012-04-12

**Authors:** Mohammed Nuruzzaman, Akhter Most Sharoni, Kouji Satoh, Ali Moumeni, Ramiah Venuprasad, Rachid Serraj, Arvind Kumar, Hei Leung, Kotb Attia, Shoshi Kikuchi

**Affiliations:** 1 Plant Genome Research Unit Agrogenomics Research Center, National Institute of Agrobiological Sciences (NIAS), Tsukuba, Ibaraki 305-8602 Japan; 2 Africa Rice Center (AfricaRice), Ibadan Station, c/o IITA, PMB 5320 Oyo Road, Ibadan, Nigeria; 3 Diversification and Sustainable Intensification of Production Systems Program (DSIPSP), International Center for Agricultural Research in the Dry Areas (ICARDA), P.O. Box 5466, Aleppo, Syria; 4 Plant Breeding, Genetics and Biotechnology Division (PBGB), International Rice Research Institute, DAPO Box 7777, 1301 Metro Manila, Philippines; 5 Graduate School of Science and Technology, Niigata University, Ikarashi-2, Niigata, 950-2181 Japan; 6 Centre of Excellence in Biotechnology Research, King Saud University, Riyadh, 11451 Kingdom of Saudi Arabia; 7 Rice Research Institute of Iran in Mazandaran, P.O. Box 145, Km8 Babol Rd., 46191-91951 Amol, Mazandaran Iran

**Keywords:** Microarray, Expression profile, Hormone, NILs, Stress

## Abstract

The *NAC* (NAM, ATAF1/2 and CUC2) genes are plant-specific transcriptional factors known to play diverse roles in various plant developmental processes. We describe the rice (*Oryza sativa*) *OsNAC* genes expression profiles (GEPs) under normal and water-deficit treatments (WDTs). The GEPs of the *OsNAC* genes were analyzed in 25 tissues covering the entire life cycle of Minghui 63. High expression levels of 17 genes were demonstrated in certain tissues under normal conditions suggesting that these genes may play important roles in specific organs. We determined that 16 genes were differentially expressed under at least 1 phytohormone (NAA, GA3, KT, SA, ABA, and JA) treatment. To investigate the GEPs in the root, leaf, and panicle of three rice genotypes [e.g., 2 near-isogenic lines (NILs) and IR64], we used two NILs from a common genetic combination backcross developed by Aday Selection and IR64. WDTs were applied using the fraction of transpirable soil water at severe, mild, and control conditions. Transcriptomic analysis using a 44K oligoarray from Agilent was performed on all the tissue samples. We identified common and specific genes in all tissues from the two NILs under both WDTs, and the majority of the *OsNAC* genes that were activated were in the drought-tolerant IR77298-14-1-2-B-10 line compared with the drought-susceptible IR77298-14-1-2-B-13 or IR64. In IR77298-14-1-2-B-10, seventeen genes were very specific in their expression levels. Approximately 70 % of the genes from subgroups SNAC and NAM/CUC3 were activated in the leaf, but 37 % genes from subgroup SND were inactivated in the root compared with the control under severe stress conditions. These results provide a useful reference for the cloning of candidate genes from the specific subgroup for further functional analysis.

**Electronic supplementary material:**

The online version of this article (doi:10.1007/s00438-012-0686-8) contains supplementary material, which is available to authorized users.

## Introduction

Most eukaryotic gene expressions rely on specific transcription factors (TFs) that modulate DNA structure in the regulatory regions of genes, and this modulation affects the activity of RNA polymerases in the initiation of transcription. Under different adverse environmental conditions, genes show specific expression patterns in accordance with their biological and physiological functions. Thus, the most important goal of developmental biology is to identify unique genes or groups of expressed genes to understand the network of genes and their expression profiles that are linked to specific organs or multiple tissues. In plants, the *NAC* gene family encodes a large number of TF genes. The NAC acronym was derived from three genes that were initially discovered to contain NAC domains: NAM (for no apical meristem), ATAF1 and -2, and CUC2 (for cup-shaped cotyledon) (Souer et al. 1996; Aida et al. 1997). The C-terminal regions of NAC proteins are highly divergent (Ooka et al. 2003) and are responsible for the diversity of the transcriptional activities exhibited by NAC proteins (Xie et al. 2000; Yamaguchi et al. 2008; Jensen et al. 2010). The earliest reports of *NAC* genes include the NAM from petunia (*Petunia hybrida*), which determines the position of the shoot apical meristem (Souer et al. 1996), and CUC2 from Arabidopsis, which participates in the development of embryos and flowers (Aida et al. 1997). Many of the NAC family proteins have been identified and are implicated in many diverse functions and cellular processes, such as hormonal signal transduction (Greve et al. 2003) and development (Peng et al. 2009), in various plant species. *AtNAC072* (*RD29*), *AtNAC019*, *AtNAC055*, and *ANAC102* from Arabidopsis (Fujita et al. 2004; Tran et al. 2004; Christianson et al. 2009); *SNAC1*, *SNAC2/OsNAC6*, *OsNAC5*, and *OsNAC10* from rice (Hu et al. 2006, 2008; Nakashima et al. 2007; Sperotto et al. 2009; Zheng et al. 2009; Jeong et al. 2010); and *BnNAC* from *Brassica napus* (Hegedus et al. 2003) were shown to be involved in responses to various environmental stresses. Lin et al. (2007) reported that the level of *OsNAC19* transcription was elevated by infection with the fungus *Magnaporthe grisea*, suggesting that *OsNAC19* is involved in the defense response of rice to *M. grisea* infection. *AtNAC2*, another stress-related *NAC* gene in Arabidopsis, functions downstream of the ethylene and auxin signal pathways and enhances salt tolerance and lateral root development when overexpressed (He et al. 2005). *NAM*-*B1*, an *NAC* gene found in wheat, is involved in nutrient remobilization from the leaves to the developing grains (Uauy et al. 2006), whereas *GRAB1* and *GRAB2* were found to interact with the dwarf geminivirus RepA protein to control geminivirus DNA replication associated with plant growth and development in wheat (Xie et al. 1999). NAC proteins generally function as transcriptional activators, and *AtNAM*, *ATAF1*, *AtNAC2*, and *AtNAC3* have been shown to act as transcriptional activators in a yeast assay system (He et al. 2005).

Drought is a major constraint for rice production in rainfed areas. Among the cereal crops, rice is very sensitive to soil water deficit and evaporative demand, with the greatest sensitivity in lowland-adapted genotypes (Parent et al. 2010). Therefore, manipulation of the genetic background of rice genotypes to improve drought tolerance is considered a promising approach for sustainable rice production in water-scarce areas (Serraj et al. 2009). Drought tolerance is a very complex trait, and a large number of genes and metabolic pathways interact in tolerance to drought stress (Lenka et al. 2011). Achieving drought tolerance requires an understanding of the underlying physiological mechanisms and the genetic control of the contributing traits in drought resistance (Bernier et al. 2009). Mechanisms of response to water-deficit stress (WDS) can be measured at many different levels, from the whole plant to the molecular level. Because stress responses are controlled by the plant genome, recent efforts have focused on the molecular response of the plant to WDS (Bray 2004). Nevertheless, information concerning the molecular aspects of the control of drought tolerance is limited. Hence, a promising approach is to use a dry-down method to examine NILs that have a common genetic background but contrasting levels of tolerance to water deficits in a long-term drought stress condition that is similar to field conditions. For gene expression analysis, the use of NILs reduces the noise of gene expression data by comparing the original cultivars. Through selection in the drought-breeding program of International Rice Research Institute (IRRI), a set of advanced backcrossed lines was developed by backcrossing Aday Selection (Aday Sel.), a drought-tolerant traditional rice variety, to IR64 (Khush et al. 2004). IR64 is the most widely grown high-yielding rice variety in the tropics and has important agronomic traits; it is resistant to blast, bacterial leaf blight, and brown planthopper, but it is susceptible to tungro disease (Khush et al. 2004) and drought stress (Guan et al. 2011). Using the IR64 as a recurrent parent, the following two NILs with contrasting drought tolerances (severe and mild) were selected (Venuprasad et al. 2007) from the IR77298-14-1-2-B family: IR77298-14-1-2-B-10 (NIL10) (highly drought tolerant) and IR77298-14-1-2-B-13 (NIL13) (susceptible to drought). These advanced backcrossed lines are considered pre-NILs because they are sister lines derived from a single family segregated for drought-tolerance phenotypes. A genetic study using 491 SSRs revealed that both NIL10 and NIL13 are 96.5–98.8 % genetically similar (Venuprasad et al. 2011). Global gene expression analyses in these NILs under drought stress conditions in the root were performed, and the gene expression data were analyzed using gene ontology profiling (Moumeni et al. 2011). A number of transcription factor gene families including NAC TF were differentially expressed in IR77298-14-1-2-B-10 in the root tissue (Moumeni et al. 2011).

The rice genome was predicted to contain 151 *NAC* genes (Nuruzzaman et al. 2010), and only a few genes have been characterized in this species (Hu et al. 2006; Jeong et al. 2010). Characterization of *NAC* family genes in rice can help us to understand the molecular mechanisms of resistance to stress and thus aid in the development of rice varieties using transgenic technology. We studied the temporal, spatial, hormonal, and drought regulation of all *OsNAC* genes in rice and used this baseline information to investigate drought-regulated *OsNAC* genes in rice NILs. To identify the putative drought-responsive genes in NILs, we used Affymetrix array data in Minghui 63 for normal growth conditions and hormone treatments as well as the Agilent 44K oligoarray system to profile the transcriptomes of *OsNAC* genes under water-deficit treatments (WDTs), and we compared the different gene expression patterns. Several specific genes, or subgroups of this gene family, revealed novel information pertaining to their role in the plant response to normal and stress conditions. Furthermore, the data revealed introgressed regions and conserved *cis*-elements in the 2-kb region upstream of the promoters of differentially expressed genes (DEGs) under drought stress conditions. To our knowledge, this is the first report that focuses on the *OsNAC* genes to identify family level expression patterns. Taken together, these results provide a solid basis for future functional genomic research of *OsNAC* genes.

## Materials and methods

### Expression profile analysis in Minghui 63

We used gene expression data for *OsNAC* genes in Minghui 63 available at NCBI-GEO (GSE19024; Wang et al. 2010, http://www.ncbi.nlm.nih.gov/), which is composed of hybridized RNA samples from 25 tissues that cover the entire life cycle of rice with the Affymetrix rice microarray. The following vegetative and reproductive tissues at different developmental stages under normal agricultural growth conditions were used for *OsNAC* gene expression profile analysis in this study: control (CK) seed, germination (72 h after imbibition); (1) calli 1, 15 days after subculture; (2) calli 2, screening stage; (3) calli 3, 5 days after regeneration; (4) seedling 1, 3 days after sowing; (5) seedling 2, root and leaf at the 3-leaf stage; (6) root, seedling with two tillers; (7) shoot, seedling with two tillers; (8) stem 1, 5 days before heading; (9) stem 2, heading stage; (10) flag leaf 1, 5 days before heading; (11) flag leaf 2, 14 days after heading; (12) leaf 1, young panicle at stage 3; (13) leaf 2, 4–5 cm young panicle; (14) sheath 1, young panicle at stage 3; (15) sheath 2, 4–5 cm young panicle; (16) panicle 1, young panicle at stage 3; (17) panicle 2, young panicle at stage 4; (18) panicle 3, young panicle at stage 5; (19) panicle 4, 4–5 cm young panicle; (20) panicle 5, heading stage; (21) stamen, just before heading; (22) spikelet, 3 days after pollination; (23) endosperm 1, 7 days after pollination; (24) endosperm 2, 14 days after pollination; and (25) endosperm 3, 21 days after pollination. The expression intensities of the samples were transformed into log_2_-based values and normalized according to the quantile method for standardization among the array data using Expander version 5.0 (Shamir et al. 2005). After normalization, the average signal value of 2 biological replicates for each sample was used for analysis. Genes were considered DEGs if they exhibited a log_2_-based ratio greater than 1 (2-fold) or less than −1 (−2-fold), and the significance of the changes in gene expression between two tissues was defined as *P* ≤ 0.05 using a paired *t* test (permutation, all; FDR collection, adjust Bonferroni method). Data processing was performed using Multi-Experimental Viewer (MEV) version 4.5 (Saeed et al. 2003).

### Phytohormone treatments

For phytohormone treatments, 20-day-old seedlings (trefoil stage) were transferred to solutions of 0.1 mM naphthalene acetic acid (NAA, a member of the auxin family), gibberellic acid (GA3), and kinetin (KT, a cytokinin). The samples were harvested at 5, 15, 30, and 60 min after treatment, and the samples subjected to the same hormone treatment at different time points were mixed together (Nuruzzaman et al. 2008). Benzothiadiazole (BTH) is a functional analog of salicylic acid (SA), and seedlings at the 4-leaf stage were treated with 5 mM BTH and were harvested after 24 h (Shimono et al. 2007). Significant DEGs in hormone-treated seedlings were identified if the expression value of a gene in a given tissue was more than twofold greater than in the control, *P* ≤ 0.05, estimated using a paired *t* test. In addition, rice seeds were grown for 3 weeks at 28 °C in the dark, and the seedlings were treated with abscisic acid (ABA, 100 μM) for 5 h (Yazaki et al. 2003). Similarly, leaf disks (6 mm in diameter) were floated on jasmonic acid (JA, 500 μM) at 25 °C under light, and samples were harvested at 6, 12, 24, and 48 h. The expression intensities in response to ABA and JA treatments are log_2_ ratio values, and genes with a threshold value greater than 1 (2-fold) or less than −1 (−2-fold) were designated as DEGs. Untreated plants were used as controls. The microarray data were downloaded from NCBI-GEO (GSE7567, GSE32634, and GSM26043 for SA, ABA, and JA, respectively).

### Plant growth and drought-treatment conditions for rice

The two NILs (IR77298-14-1-2-B-10 and IR77298-14-1-2-B-13) and the IR64 line were chosen for this study due to their different yields under WDTs. The two NILs were derived from the IR77298-14-1-2 family, and this family was developed at IRRI by backcrossing Aday Selection × IR64 (Khush et al. 2004). IR77298-14-1-2-B-10 and IR77298-14-1-2-B-13 also differ in their tolerance for drought (Venuprasad et al. 2007). IR77298-14-1-2-B-10 (NIL10) is a high-yielding drought-tolerant NIL (DTN), whereas IR77298-14-1-2-B-13 (NIL13) is a drought-susceptible NIL (DSN) under severe and mild stress conditions. In contrast, NIL10 and NIL13 exhibit similar yield potentials under normal growth conditions.

Plant materials were grown in PVC pipe columns measuring 1.05 m high and with an 18-cm diameter that were filled with 10 kg of a soil and sand mix (2 parts soil: 1 part sand). The plants were adequately fertilized and grown under controlled conditions. Initially, the experiment was conducted in the greenhouse and was moved to natural conditions before imposing the drought stress. The soil in the pots was saturated and covered with transparent plastic covers with an opening in the center to facilitate planting. A feeder pipe was inserted for watering the pots. In each pot, five germinated seeds were transplanted, and the plants were reduced to two plants per pot at the 3-leaf stage. For this experiment, there were four replications, and a randomized complete block design was used.

The pots were watered twice daily to keep the soil saturated. The day before the start of the progressive soil-drying process, the soil in each pot was saturated with water. Stress was imposed by initiating a soil dry-down protocol starting 35 days after sowing. The dry-down process continued until the pot reached the target fraction of transpirable soil water (FTSW) (Sinclair and Ludlow 1986). The weight of each pot was measured every day during the dry-down period to estimate the transpiration. The WDTs were a control (1.0 FTSW) consisting of well-watered plants and soil that was kept saturated throughout the experiment, and two drought stresses including severe (0.2 FTSW) and mild drought stress conditions (0.5 FTSW). No water was applied to the soil during the dry-down period. We maintained the target FTSW in all pots until harvesting the plants.

### RNA extraction

Total RNA was extracted from 10-mm samples of the root tip, leaf, and panicle of plants from all treatment conditions, i.e., 1.0, 0.5 and 0.2 FTSW at the reproductive stage. The RNA samples were prepared in triplicate using an RNeasy Maxi kit (Qiagen). The concentration and quality of the microarray samples were examined using a Nanodrop spectrophotometer (Nanodrop ND-1000; Nanodrop Technologies) and a BioAnalyzer (G2938A; Agilent Technologies). For the microarray experiments, 60 independent RNA samples from the root, leaf, and panicle were prepared.

### Microarray experiment and data analysis

In this study, the probe and array designs were performed using the eArray version 4.5 supplied by Agilent Technologies (https://earray.chem.agilent.com/earray/), and 43,494 probes were selected for this custom array. The 43,494 probes, four sets in total (4 × 44K microarray formats), were blotted on a glass slide (25 × 75 mm) at Agilent Technologies in triplicate. Cyanine 3 (Cy3)- or cyanine 5 (Cy5)-labeled cRNA samples were synthesized from 850 ng total RNA using a Low Input RNA labeling kit (Agilent Technologies) in accordance with the manufacturer’s instructions. Transcriptome profiles specific to stressed plants were examined by the direct comparison of transcription activities between stressed conditions and non-stressed (control) plants on the same oligoarray. Hybridization solution was prepared containing 825 ng of each of the Cy3- and Cy5-labeled cRNA preparations using an in situ Hybridization Kit Plus (Agilent Technologies). Hybridization and washing of the microarray slides were performed in accordance with the manufacturer’s protocols. After washing, the slide image files were produced using a DNA microarray scanner (G2505B; Agilent Technologies).

The image files of the slides were processed using Feature Extraction version 9.5 (Agilent Technologies). The Cy3 and Cy5 signal intensities were normalized using rank-consistency filtering and the LOWESS method. The signal intensities of all samples were transformed into log_2_-based ratio (stressed sample/control-non-stressed sample) and were normalized as described above. A gene was designated ‘expressed’ if the mean signal intensity of the gene was >6 in least at one of the conditions; otherwise, the gene was considered not expressed. To identify DEGs, a paired *t* test was performed as described above, and only genes that exhibited a *P* value less than 0.05 with more than a twofold increase in transcription levels compared with the control were considered significant in specific tissues under severe and mild stress conditions. The probe arrangement of the array data (platform number GPL7252, GSE30463) is available at NCBI-GEO (Barrett et al. 2007).

### Semiquantitative RT-PCR

The same RNA samples (root, leaf, and panicle) as those used for the microarray were used for the RT-PCR experiments. The RNA samples were pretreated extensively with RNase-free DNase I (Invitrogen) to eliminate any contaminating genomic DNA. The first-strand cDNA was synthesized from 1 μg of total RNA in a 20-μl reaction volume using Superscript II Reverse Transcriptase (Invitrogen), and 2 μl of the reaction mixture was subsequently used for RT-PCR in a 50-μl reaction volume. To synthesize first-strand cDNA from the DNase I-treated total RNA, semiquantitative RT-PCR was performed using SuperScriptII Reverse Transcriptase in accordance with the manufacturer’s instructions. The RNA samples for hybridization and RT-PCR were the same. Approximately 1/20 of the first-strand cDNA generated from 1 μg total RNA was used as a template for PCR in a reaction volume of 50 μl with rTaq DNA polymerase (Takara). The RT-PCR runs consisted of 25–38 cycles, depending on the linear range of PCR amplification for each gene. Each PCR was performed in triplicate in an ABI 9700 Thermocycler (Applied Biosystems). The program consisted of incubation at 94 °C for 1 min, at 55 °C for 50 s, and at 72 °C for 1 min. The rice *actin* gene (LOC_Os05g36290) was used as an internal control for RT-PCR (Nuruzzaman et al. 2010; Sharoni et al. 2011), and the primers are listed in Supplementary Table 1.

## Results

### Global *OsNAC* gene expression patterns under normal conditions in Minghui 63

**Fig. 1:**
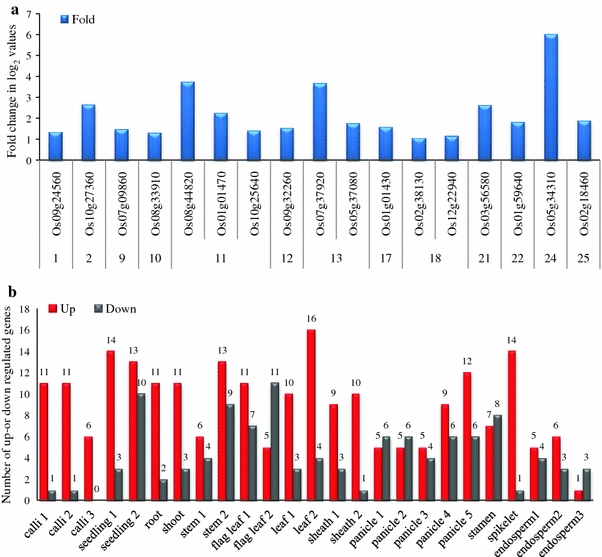
**a** Tissue-specific up-regulated genes in Minghui 63, the name of the tissues and the number of genes are presented to the right of the graph. The fold changes of log_2_ values are shown on the *Y* axis, and the different developmental stages and genes are indicated on the *X* axis. **b** The number of up- or down-regulated genes in different tissues under normal growth conditions in Minghui 63 is shown. The number of genes (up or down) is shown on the *Y* axis, and the different developmental stages are indicated on the *X* axis. Differentially up- or down-regulated genes at the different developmental stages are presented in Supplemental Table 2

To provide a global overview of the *OsNAC* transcriptome, specific tissues and developmental stages were selected to obtain an overview of the basal expressions levels for comparison with the results from the normal growth conditions. Detailed information on the selected tissues can be found in “Materials and methods”. The Affymetrix microarray data that we used revealed the expression profiles of various genes (Nuruzzaman et al. 2008; Dai et al. 2009; Ye et al. 2009). Probes for 132 of the 151 *OsNAC* genes were identified in the Affymetrix microarray. For convenience, the “LOC_” prefix has been omitted from the Michigan State University (Osa1 Ouyang et al. 2007) locus IDs in the manuscript. The *OsNAC* genes that exhibited differential expression during various stages of development compared to the seed (control) were selected for analysis. Detailed *P* values and differential expression log_2_-fold change values are shown in Supplementary Table 2. According to the log_2_ signal values, most of the *OsNAC* genes were expressed in at least one of the 25 investigated tissues. Based on the gene structure, the *OsNAC* genes were classified into 16 subgroups in rice (Nuruzzaman et al. 2010). It is not practical to describe the expression of all the *OsNAC* genes in all the examined tissues; therefore, we have selected subgroups for the analysis and discussion below. Of the 16 subgroups, 2 genes, TIP and SNAC (TIP: *turnip crinkle* virus interacting protein, SNAC: stress-associated NAC), were expressed predominantly in the root and flag leaf 2, as well as in the endosperm 1 and 2. A total of three genes assigned to subgroup NAM/CUC3 (NAM, no apical meristem) were up-regulated preferentially in the seedling 1, root, stem 2, leaf 2, and sheath 1, and three more genes belonging to subgroups SND and ONAC4 (SND: secondary wall-associated NAC domain) were expressed predominantly in the seedling 2 and calli 2 (Supplementary Table 2). Interestingly, *Os01g29840* (subgroup NAM/CUC3) was activated in the majority of the studied tissues. Genes (17) belonging to different subgroups, such as TIP (*Os08g44820*), NAM/CUC3 (*Os09g32260* and *Os01g01470*), ANAC34 (*Os08g33910* and *Os03g56580*), SNAC (*Os01g01430*, *Os05g34310*, and *Os07g37920*), ONAC4 (*Os02g38130*, *Os10g25640*, and *Os10g27360*), ONAC2 (*Os07g09860* and *Os02g18460*), ONAC3 (*Os01g59640* and *Os12g22940*), ONAC7 (*Os05g37080*), and NEO (*Os09g24560*), exhibited high expression patterns in a number of tissues in particular, and the highest expression levels (2-fold or more) were observed in the calli, stem, flag leaf, leaf, panicle, stamen, spikelet, and endosperm (Fig. 1a, Supplementary Table 2). A biphasic expression pattern was also observed; for example, *Os11g03370*, *Os12g05990*, and *Os09g24560* genes were up-regulated in the vegetative stages but down-regulated or not differentially expressed in the reproductive stages. Similarly, *Os12g43530*, *Os01g71790*, and *Os05g37080* genes were up-regulated in the reproductive stages but down-regulated in the vegetative stages. As shown in Fig. 1b, a large number of up-regulated genes were found (e.g., leaf 2, seedling 1, and spikelet) in Minghui 63 in most of the developmental stages; in contrast, the number of down-regulated genes was much greater in the flag leaf 2, panicle 1 and 2, stamen, and endosperm 3 in the reproductive stages. In the vegetative stages, 53 *OsNAC* genes were up-regulated, and 21 genes were down-regulated. In contrast, 59 genes were up-regulated, and 32 genes were down-regulated in Minghui 63 in the reproductive organs (Fig. 1b). These results reveal that the differentially regulated *OsNAC* genes exhibit high variations in the reproductive stages compared with the vegetative stages, implying the activation of different genes at different developmental stages.

### The responses of *OsNAC* genes to naphthalene acetic acid (NAA), gibberellic acid (GA3), kinetin (KT), salicylic acid (SA), abscisic acid (ABA), and jasmonic acid (JA) treatments

**Fig. 2:**
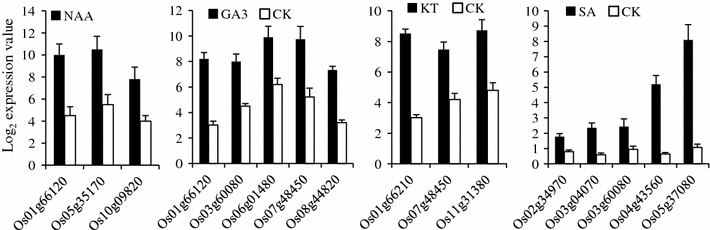
Histogram showing up-regulated *OsNAC* genes in seedlings subjected to treatment with four phytohormones (*NAA* naphthalene acetic acid, *GA3* gibberellic acid, *KT* kinetin, *SA* salicylic acid). The *X* axis represents the differentially expressed genes. The *Y* axis represents the log_2_ expression values. The treatment is indicated at the *top of each diagram*. The *error bars* represent standard deviations. *CK* control or untreated samples

Genetic factors, external environmental factors, and hormones inside the plant influence the growth and development of a plant. Phytohormones play critical roles in the plant life cycle during growth and development. To investigate the *OsNAC* genes that respond to phytohormone treatment, microarray analysis was performed. In total, 16 DEGs were identified following treatments with one or more of the phytohormones NAA, GA3, KT, and SA in seedlings. They were compared to the control (untreated samples), and the fold change log_2_ values with respect to the control are shown in Supplementary Table 3. Among these DEGs, 14 genes belonging to different subgroups (e.g., TIP, SND, and SNAC) were up-regulated, whereas 2 genes were down-regulated (Fig. 2). Interestingly, *Os01g66120* and *Os07g48450,* which belong to the SNAC subgroup, were up-regulated specifically by two or more of the NAA, GA3, and KT treatments but down-regulated in the control (Fig. 2). Similarly, at the 3-week-old seedling stage in Nipponbare rice, four genes were up-regulated under ABA (100 μM) treatment, compared with the control, whereas one gene (*Os06g04090*, SND) was activated when treated with JA (500 μM) (Supplementary Table 4).

### Differential expression profiles in NILs

In this study, we examined the effects of two drought treatments on transcriptome changes in the root, leaf, and panicle stages of the tolerant line IR77298-14-1-2-B-10 (NIL10), the susceptible line IR77298-14-1-2-B-13 (NIL13) and the control IR64. For the NILs, gene identification and the determination of gene expression patterns are important in understanding how the plant responds under drought-stress conditions. The three WDTs used were 0.2 FTSW (severe stress), 0.5 FTSW (mild stress), and 1.0 FTSW (control). We focused on the three genotypes (NIL10, NIL13 and IR64) and a number of points, such as DEGs (adj. *P* ≤ 0.05, at 2- and 4-fold) identified in NIL10 versus NIL13 or IR64; the number of genes that were specifically expressed in tolerant NIL, in both NILs, and in individual and multiple tissues under both WDTs; and DEGs of different subgroups of the *OsNAC* genes, as compared with normal and 0.2 FTSW. Finally, we identified the most promising candidate drought responsive *OsNAC* genes in tolerant lines for further functional analysis. Expression profiling and analysis indicated the involvement of this large gene family in a number of signaling pathways triggered by drought stress and a possible role for these genes in plant development.

Out of 151 genes, we identified 131 *OsNAC* genes in our 44K array data. Among these genes, 103 were differentially expressed (up- or down-regulated) under both WDTs in different tissues (Supplementary Table 5). We found that the tolerant line NIL10 demonstrated the highest number of up-regulated genes (followed by IR64 and NIL13) in the root, leaf, and panicle under 0.2 FTSW and 0.5 FTSW conditions, with the exception of the leaf under 0.5 FTSW (Supplementary Table 6). In response to 0.2 FTSW, the tolerant line exhibited the highest number of up-regulated genes (34) in the leaf. Hence, despite their common genetic background as backcrossed progeny from Aday Sel × IR64, the two NILs demonstrated distinct differences in their gene expression patterns in response to WDTs. The gene expression profiles among Minghui 63, IR64, and NILs were compared in the root, leaf, and panicle under normal and drought stress condition. The majority of the *OsNAC* genes exhibited similar expression levels (average log_2_ values) in Minghui 63 and in IR64 (data not shown) in the three tissues. However, a number of the genes exhibited slightly different expression intensities because three samples had been taken from two platforms of microarray analysis, but the same genes showed differential expression in the tolerant line or in both NILs under both WDTs (Table 1). Furthermore, we identified the DEGs in both NILs under 0.2 FTSW and 0.5 FTSW conditions in the root, leaf, and panicle, and we compared their expression profiles under normal growth conditions and after treatment of the seedlings with different hormones, as described below.

**Table 1:** The basal and stress-response expression (severe or mild drought) of *OsNAC* genes in different rice genotypes

Tissue	Gene ID in MSU Osa1	Conventional name of the gene and its function	Normal expression intensities (log_2_)		Gene response in NILs under drought stress (DEG)	
			Minghui 63	IR64	NIL10	NIL13
Root severe	*Os07g04560*	NAC domain-containing protein 94	11.36	12.19	1.10	–
	*Os07g27330*	No apical meristem protein	2.71	3.06	−1.28	–
Leaf severe	*Os02g12310*	NAC domain-containing protein 18	2.81	2.82	4.55	3.62
	***Os03g60080*** **/** ***SNAC1***	**Stomata closed, higher seed setting**	7.06	8.88	3.82	2.25
Leaf mild	*Os06g04090*	NAM protein	8.98	9.80	1.0	–
	*Os07g12340*	NAC domain-containing protein 67	9.54	10.85	1.05	0.71
	***Os03g60080*** **/** ***SNAC1***	**Stomata closed, higher seed setting**	7.06	8.88	2.08	1.89
Panicle severe	*Os09g38000*	Ortholog of ANAC086	5.41	5.11	2.94	2.47
	*Os05g34310*	NAC domain-containing protein 94	5.80	4.71	2.09	1.35
	*Os07g12340*	NAC domain-containing protein 67	8.17	9.18	1.90	1.90
	***Os11g03300/OsNAC10***	**Root, panicle, drought, salt, ABA**	10.02	10.93	4.56	3.66
	*Os02g38130*	Ortholog of ANAC044	7.02	8.46	1.42	1.05
	*Os06g15690*	Ortholog of ANAC008	9.16	8.92	1.08	0.77
	*Os09g12380*	No apical meristem protein	3.67	3.47	1.81	
Panicle mild	***Os01g66120/SNAC2/6***	**Salt, drought, disease resistance**	10.47	11.28	3.94	2.17
	***Os11g03300/OsNAC10***	**Root, panicle, drought, salt, ABA**	10.02	10.19	4.84	4.25

The genes in bold have been characterized in rice, and the fold changes (log_2_ ratio values) are shown in Supplementary Table 5

*DEG* differentially expressed genes (DEGs) up-/down-regulated, – not differentially expressed

### *OsNAC* gene expression in the root common to both NILs under severe and mild stress conditions

**Fig. 3:**
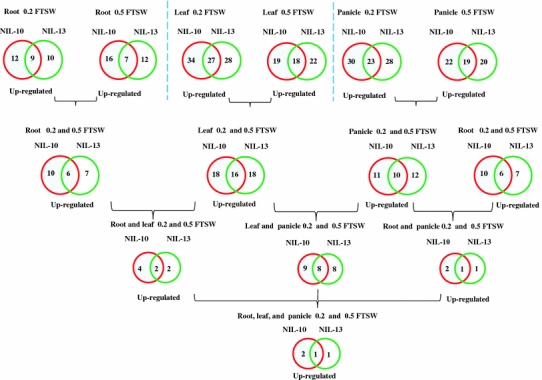
Up-regulated overlapped and specific genes were detected in R77298-14-1-2-B-10 (NIL10) and IR77298-14-1-2-B-13 (NIL13) under 0.2 and 0.5 FTSW conditions in the root, leaf, and panicle

In this study, gene expression profiles were compared between the drought-tolerant and drought-susceptible lines. Such NILs are very useful in physiological and genetic studies. We compared the gene expression profiles between the drought-tolerant and drought-susceptible lines and observed that genotypes responding differentially to drought stress exhibited variations in gene expression in particular tissues and that a portion of the differences relate to drought tolerance or drought response. Spollen et al. (1993) reported that mild osmotic stress rapidly inhibits the growth of the leaves and stems but the roots continue to elongate; thus, the root architecture is a key trait for dissecting genotypic differences in rice responses to drought tolerance. Therefore, to identify up-regulated genes in IR77298-14-1-2-B-10 (NIL10) versus IR77298-14-1-2-B-13 (NIL13), the analysis of genes in both NILs and in the individual strains is of interest for identifying the most promising candidate genes under both WDTs in the root, leaf, and panicle stages. This analysis is a starting point for further elucidating the role of a single gene in the stress response and will be of great value in crop engineering. Many genes respond to a single treatment but are differentially up-regulated in response to several conditions. Hence, we were interested in comparing the effects of different stresses. In this study, a gene was considered differentially expressed in one line and in a specific tissue if that gene was not differentially expressed or demonstrated a low-level of expression in the other line under severe or mild stress conditions. We investigated nine common genes in NIL10 and NIL13 that were expressed in the root under 0.2 FTSW (Fig. 3, Supplementary Table 7). Of the nine genes, six were assigned to the TIP, SND, and ONAC2 subgroups. In NIL10, we identified three specific genes (*Os05g34830*, *Os07g04560*, and *Os10g38834*) that were expressed in the root under 0.2 FTSW, but their expression patterns did not change in NIL13 under 0.2 FTSW. Similarly, in NIL13, *Os10g27360* (subgroup ONAC4) was specifically expressed in the root under 0.2 FTSW, whereas it was not differentially expressed or there was no change in its expression in the same tissue in NIL10. We discovered seven common genes in the roots of NIL10 and NIL13 under 0.5 FTSW (Fig. 3, Supplementary Table 7). We determined that nine genes (*Os02g34970*, *Os02g36880*, *Os02g57650*, *Os03g03540*, *Os05g10620*, *Os05g34830*, *Os07g04560*, *Os10g27360*, and *Os10g38834*) expressed specifically in NIL10 were not differentially expressed in NIL13 under 0.5 FTSW in the root (Supplementary Table 7). In NIL13, five genes (*Os03g42630*, *Os09g38010*, *Os10g09820*, *Os11g31380*, and *Os11g05614*) were up-regulated, whereas these genes were not differentially expressed or exhibited no change in expression in NIL10 under 0.5 FTSW conditions in the root. Thus, it appears that the expression of these genes is line- and tissue-specific under 0.2 FTSW and 0.5 FTSW conditions (Supplementary Table 7). We noted that NIL10 and NIL13 shared six up-regulated genes under all WDTs in the root (Fig. 3, Supplementary Table 7). Under both WDTs in the root, four genes (*Os02g57650*, *Os05g34830*, *Os07g04560*, and *Os10g38834*) were expressed specifically in NIL10. The expression levels of these specific genes were compared under normal growth conditions and different hormone treatments in Minghui 63. Only *Os03g42630* was up-regulated in the root under normal growth conditions. In total, eight genes (e.g., *Os07g04560* and *Os10g38834*) were not differentially expressed or were expressed at low-levels in all tissues analyzed in Minghui 63. Under 0.2 FTSW and 0.5 FTSW conditions, *Os05g34830* (SNAC) and *Os02g34970* (ONAC6) were NIL10 specific in the root and were activated by ABA and SA treatments. *Os10g09820* and *Os11g31380* (ONAC5 and ONAC1) were expressed specifically in NIL13 under mild stress and were induced by NAA and KT. Furthermore, two genes (*Os06g04090* and *Os08g44820*, subgroups SND and TIP) that were common to NIL10 and NIL13 under both FTSW conditions were up-regulated in seedlings by JA and GA3 treatments. Interestingly, *Os05g34830* (SNAC) is hormone (ABA) specific, root tissue specific and NIL10 specific under both WDTs, and its expression level is fivefold higher in NIL10 compared with NIL13 and IR64 at 0.5 FTSW; however, according to the Minghui 63 data, *Os05g34830* demonstrated lower expression levels in the root. These data suggest the specific NIL or different stresses may alter the tissue specificity of gene expression.

### *OsNAC* gene expression overlaps in the leaf in tolerant NIL10 and susceptible NIL13 under severe and mild stress conditions

A larger number of up-regulated genes (27) were detected in NIL10 and NIL13 under 0.2 FTSW in the leaf (Fig. 3, Supplementary Table 7). Under stress conditions, seven genes (*Os01g01470*, *Os01g29840*, *Os01g28050*, *Os07g48550*, *Os11g04960*, *Os11g05614*, and *Os11g31380*) were up-regulated specifically in the NIL10 leaf. For example, at 0.2 FTSW, *Os11g31380* (ONAC1) expression in the leaf was activated approximately eightfold in NIL10 but was not differentially expressed in NIL13 (Supplementary Table 7). We determined that two genes (*Os02g42970* and *Os11g07700*) were expressed specifically in NIL13, but they were inactivated in NIL10 under 0.2 FTSW in the leaf. Of the genes up-regulated in the leaf under 0.5 FTSW, 18 were common to both lines (Fig. 3, Supplementary Table 7). We demonstrated that two genes (*Os06g04090* and *Os07g12340*, SND and SNAC) were highly activated in NIL10 specifically at 0.5 FTSW in the leaf, whereas neither gene was differentially expressed in NIL13. Similarly, four genes (*Os03g03540*, *Os03g21060*, *Os05g10620*, and *Os10g33760*) were activated specifically in NIL13 under 0.5 FTSW but were not differentially expressed in NIL10 (Supplementary Table 7). Under both FTSW conditions, two genes (*Os06g04090* and *Os11g05614*) were up-regulated specifically in NIL10. With the exception of *Os01g29840* and *Os06g04090*, the specific genes mentioned above were up-regulated in the leaf 1 (very similar to drought stress tissue) under normal conditions in Minghui 63. In total, four specific genes (*Os01g01470*, *Os01g29840*, *Os02g42970*, and *Os10g33760*) under both WDTs were up-regulated at one or more reproductive stages in Minghui 63. There were eight common genes (*Os01g66120*, *Os02g34970*, *Os03g21030*, *Os03g60080*, *Os05g34830*, *Os06g04090*, *Os07g48450*, and *Os08g44820*) activated in the leaf under both WDT conditions in both NILs, and these genes were up-regulated by NAA, SA, ABA, GA3, and JA. Of these eight genes, four were common to the root and were specific to the NILs, as described above. For example, *Os07g48550* and *Os06g04090* were up-regulated by ABA and JA, which were NIL10 specific under 0.2 FTSW and 0.5 FTSW, respectively. Interestingly, *Os06g04090* was expressed in the root in NIL10 and NIL13, but in the leaf, this gene was NIL10 specific. Similarly, *Os05g34830* (SNAC) was specifically activated in NIL10 under 0.2 FTSW in the root but was activated in the leaf in both NILs under both WDTs. We speculate that some genes (e.g., *Os05g34830*) are specific to NILs and to tissue(s) under different drought conditions and hormone treatments, and we suggest that the stress-induced transcriptome changes of these genes are constitutive.

### Overlap of *OsNAC* panicle gene expression in tolerant NIL10 and susceptible NIL13 under severe and mild stress conditions

In the panicle, under 0.2 FTSW, 23 up-regulated genes were common to NIL10 and NIL13 (Fig. 3, Supplementary Table 7). A number of genes up-regulated in the leaf and panicle in both NILs under 0.2 FTSW were assigned to subgroups SNAC and NAM/CUC3. We determined that seven genes (*Os02g34970*, *Os03g12120*, *Os03g59730*, *Os06g15690*, *Os08g06140*, *Os08g33670*, and *Os09g12380*) were specifically up-regulated in the NIL10 panicle under 0.2 FTSW. Similarly, five genes (*Os03g61319*, *Os10g26240*, *Os11g03310*, *Os11g31380*, and *Os12g03050*) were up-regulated specifically in the NIL13 panicle under 0.2 FTSW (Supplementary Table 7). These 12 specific genes (with the exception of *Os03g59730*) under 0.2 FTSW were not up-regulated in the panicle 1 (similar to stress tissue) under unstressed conditions in Minghui 63. Of these 12 genes, 3 genes (*Os02g34970*, *Os03g59730*, and *Os12g03050*) were up-regulated in most of the reproductive tissues; however, the remaining 9 genes were not differentially expressed or down-regulated in any of the tissues under unstressed conditions. In the panicle under 0.5 FTSW, 19 up-regulated genes were common to both NILs (Fig. 3, Supplementary Table 7). In NIL10 under 0.5 FTSW conditions, three genes (*Os01g70110*, *Os04g43560*, and *Os08g10080*) were up-regulated specifically in the panicle, and these genes were not differentially expressed in NIL13. Similarly, the *Os08g42400* gene was up-regulated specifically in the NIL13 panicle under 0.5 FTSW; however, it was not differentially expressed in NIL10 (Supplementary Table 7). *Os08g10080* gene expression was similar to its expression in normal growth tissue, whereas *Os04g43560*, *Os04g43560*, and *Os08g42400* were up-regulated in one or more reproductive stages. Out of four genes specifically expressed under 0.5 FTSW in both NILs, *Os02g34970* and *Os04g43560* genes were NIL10-specific and were activated by SA. The *Os04g43560* gene was activated in the root and panicle in NIL10 specifically under 0.5 FTSW. During the panicle stage, our results suggest that more genes were involved in NIL10 responses compared to NIL13 under 0.2 FTSW. In the panicle, under both WDTs, the two NILs shared ten common genes (Fig. 3, Supplementary Table 7). These ten genes were expressed under at least one condition in the root and leaf. Of these ten genes, four genes (*Os01g66120*, *Os03g21030*, *Os03g60080*, and *Os05g34830*) were activated in the seedlings treated with NAA, ABA, and GA, which were also activated in the leaf tissue under both WDTs, as mentioned previously. These drastic expression changes under the different drought stress conditions in NILs suggest the recruitment of normally silent or low expressed genes for emergencies.

### *OsNAC* gene expression in two NILs and under two WDTs in root versus leaf and panicle, and leaf versus panicle

**Fig. 4:**
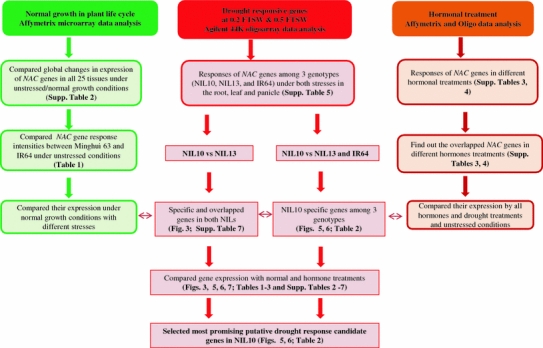
Diagram showing the microarray data and presenting the most promising candidate gene selection process under different drought conditions in the tolerant IR77298-14-1-2-B-10 (NIL10) line among all genotypes. The susceptible line NIL13 = IR77298-14-1-2-B-13 and IR64 = control

We determined that two genes were up-regulated in both lines under both WDTs in both the root and leaf (Fig. 3, Supplementary Table 7). *Os05g34830* and *Os06g04090* were expressed specifically in the NIL10 under both WDTs in the root and leaf (Supplementary Table 7). Interestingly, one gene (*Os08g44820*, TIP) was up-regulated in the root but not in the panicle under both WDTs (Supplementary Table 7). Complex genetic networks function during the development of each organ in plants, and substantial gene-expression overlaps exist between the developmental pathways and the stress-response pathways (Cooper et al. 2003). Under both WDTs, eight genes that were up-regulated in both the leaf and panicle overlapped in NIL10 and NIL13. Of these eight genes, five (*Os03g60080/SNAC1*, *Os05g34830*, *Os01g66120/SNAC2/6*, *Os11g03300/SNAC10*, and *Os12g03040*) were assigned to the SNAC subgroup, and another three genes (*Os02g36880*, *Os03g21030*, and *Os08g44820*) were assigned to the NAM/CUC3 and TIP subgroups (Fig. 3, Supplementary Table 7). The expression intensity of a number of genes in NIL10 was much greater than in NIL13, and most of the genes were assigned to the SNAC and NAM/CUC3 subgroups and were up-regulated by different hormone treatments. These findings indicate that five genes (e.g., *Os02g34970*, *Os05g34830*, *Os06g04090*, *Os07g48550*, and *Os04g43560*) from the ONAC6, SNAC, SND, NAM/CUC3, and NAC22 subgroups, respectively, were specifically expressed in NIL10 in the root, leaf, and panicle, but they were not differentially expressed in NIL13 under the two stress conditions. All five of these genes were induced by SA, ABA, and JA treatments. Therefore, the expression of these genes might be transcriptionally regulated in a variety of cell types at different developmental stages and in response to various environmental stresses. The different array data suggest that the candidate genes that were most responsive to drought were selected by following method (Fig. 4). Results from this series of studies revealed that a small number of genes contribute to drought tolerance. Because the NILs are field-proven genetic stocks that are adapted to the rainfed and upland rice-production environment, the results are likely to have agronomic relevance. The majority of the genes mentioned in this study exhibited higher expression levels in the root, leaf, and panicle under either WDT in the tolerant line compared with the susceptible line.

### Selection of the most promising putative drought responsive candidate genes and subgroups

**Fig. 5:**
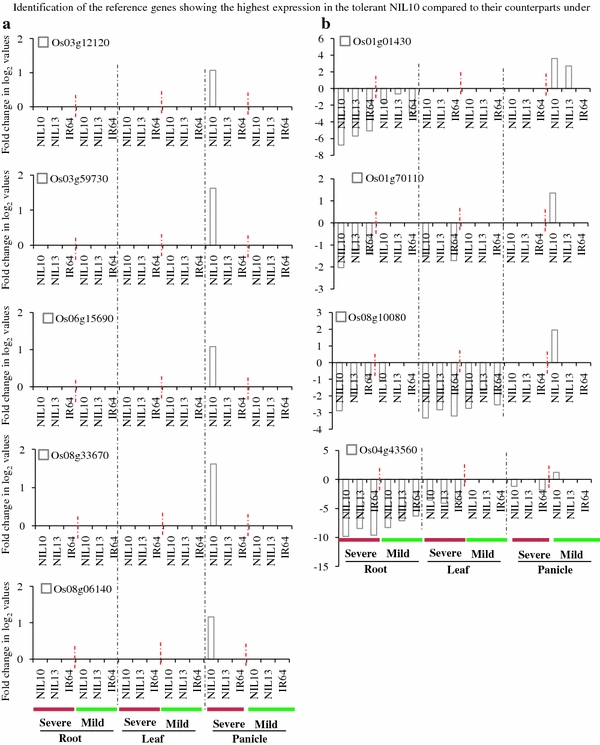
Identification of the reference genes showing the highest expression in the tolerant NIL10 compared to their counterparts under **a** severe stress in the panicle and **b** mild stress in the panicle. The fold changes in log_2_ values are shown on the *Y* axis, and the different genotypes and stress conditions are indicated on the *X* axis. NIL10 = IR77298-14-1-2-B-10 and NIL13 = IR77298-14-1-2-B-13

**Fig. 6:**
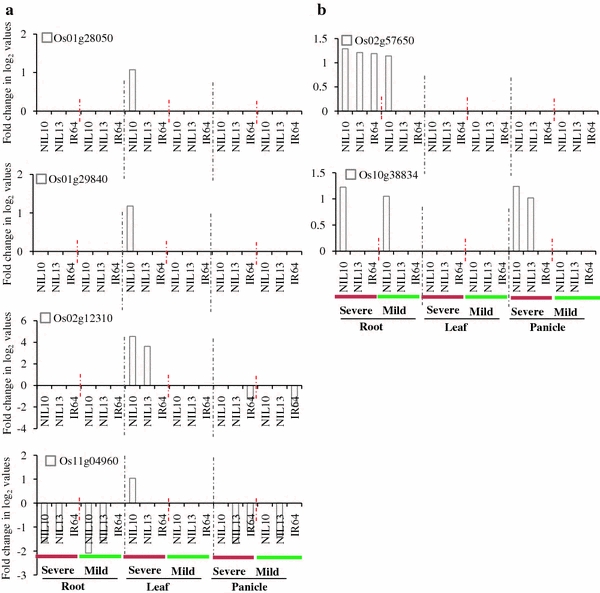
Identification of the reference genes showing the highest expression in the tolerant NIL10 compared to their counterparts under severe and mild stress **a** in the root and panicle and **b** in the leaf and panicle tissues. The fold changes in log_2_ values are shown on the *Y* axis, and the different genotypes and stress conditions are indicated on the *X* axis. NIL10 = IR77298-14-1-2-B-10 and NIL13 = IR77298-14-1-2-B-13

**Fig. 7:**
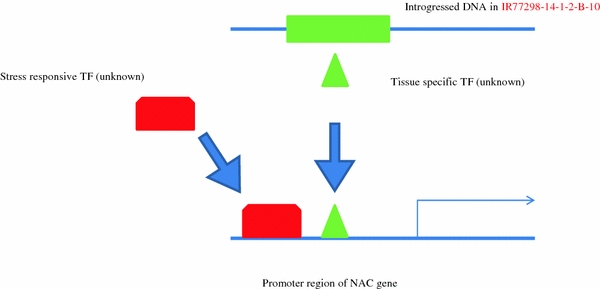
Genotyping and phenotyping models of near-isogenic lines (NILs)

**Fig. 8:**
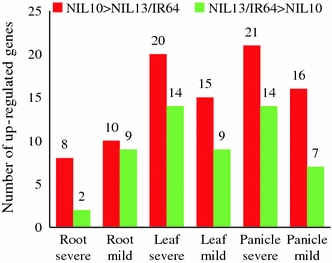
The expression intensity of up-regulated genes was higher in IR77298-14-1-2-B-10 than in IR77298-14-1-2-B-13/IR64 in all tissues under severe and mild stress conditions. The *X* axis represents the selected tissues and the *Y* axis represents number of up-regulated genes. The number of genes is indicated at the top of each diagram

**Fig. 9:**
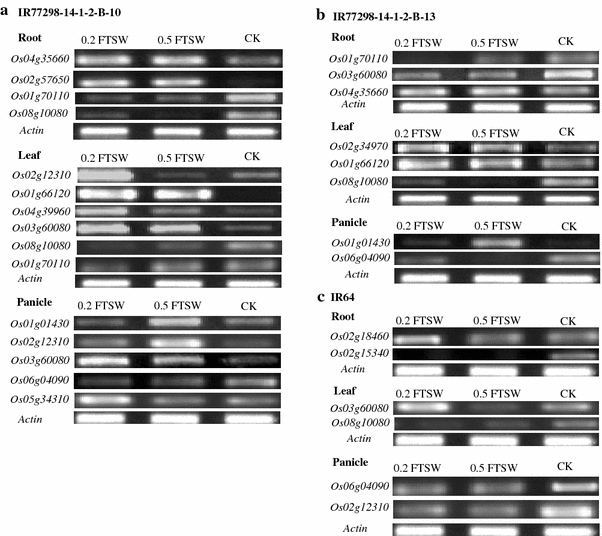
Expression profiles of 12 differentially expressed *OsNAC* genes in the root, leaf, and panicle under severe and mild drought stress in three rice genotypes. **a** Root, leaf, and panicle in IR77298-14-1-2-B-10, **b** root, leaf, and panicle in IR77298-14-1-2-B-13, and **c** root, leaf, and panicle of the IR64. Genotype names are indicated at the *top of each gel image*. *CK* control. The fold changes in log_2_ signal values of the genes are shown in Supplementary Table 5

To identify putative candidate genes that are responsible for drought tolerance in the tolerant NIL10, this study focused on DEGs that exhibited high levels of expression in response to two WDTs in the tolerant line versus the susceptible line and IR64. In the tolerant line NIL10, nine genes (*Os01g01430*, *Os01g70110*, *Os03g12120*, *Os03g59730*, *Os04g43560*, *Os06g15690*, *Os06g06140*, *Os08g10080*, and *Os08g33670*) exhibited higher expression levels (≥2-fold) in the panicle than in the root and leaf under 0.2 FTSW and 0.5 FTSW (Fig. 5a, b). In the leaf under 0.2 FTSW, four genes (*Os01g28250*, *Os01g29840*, *Os02g12310*, and *Os11g049060*, subgroups ONAC2, NAM/CUC3, SNAC, and ONAC5, respectively) demonstrated higher expression levels in NIL10 compared to the control (Fig. 6a). *Os02g57650* (TIP) and *Os10g38834* (SND) were highly activated in the root and panicle, respectively, under both WDTs in the tolerant line (Fig. 6b). Based on these findings from the promoter transactivation experiments, we proposed a model for the role of the expression of specific genes in the transcriptional network under stress condition in the NILs (Fig. 7). Of the two NILs, NIL10 has the larger number of introgressions. We assume that the IR64 background introgressions bind with unknown tissue-specific transcription factors in the promoter region of *NAC* genes under stress conditions and activate the genes in the tolerant line, whereas these factors or introgressions are absent in the susceptible line. All of the above genes (with the exception of *Os03g59730*, *Os08g10080*, and *Os01g29840*) were expressed at a low level or were down-regulated in all tissues under normal growth conditions, whereas *Os06g04090* and *Os04g43560* were up-regulated with JA and SA treatment in the seedlings. A small number of genes (e.g., *Os03g59730*, Table 2) were up-regulated in the reproductive tissues in NIL10 and in Minghui 63 under both stress and normal conditions, but the expression intensities were much greater in NIL10 (data not shown) and the up-regulation in IR64 was not significant. Therefore, we infer that the above genes might play functional roles in the specific tissue under both drought stress conditions in the tolerant line. Further investigation is required to distinguish these possibilities. Table 2 shows that in total, 17 genes, including the above-mentioned genes, were up-regulated under both WDTs in the NIL10; however, these genes were down-regulated or their expression levels did not change in NIL13 and IR64, and some genes (e.g., *Os06g04090* and *Os04g43560*) were up-regulated under normal conditions and following hormone treatments. We determined that three genes overlapped between the leaf and panicle, but none of the genes overlapped in the root and leaf or the root and panicle. In addition, following treatment with different hormones, one gene (*Os12g03040*) was up-regulated in all the tissues in Minghui 63, suggesting that *Os12g03040* is a housekeeping gene (Table 2). We observed that in the root, leaf, and panicle tissues, the highest expression intensity of a number of the up-regulated genes was found in IR77298-14-1-2-B-10, followed by IR77298-14-1-2-B-13 and IR64 (Fig. 8). In addition, analysis of the DEGs (up-regulated) found exclusively in the tolerant IR77298-14-1-2-B-10 line is useful for the identification of a specific subgroup(s) of *NAC* genes involved in severe drought stress compared with normal growth conditions in IR64 or Minghui 63. In one case, a large difference in DEGs (up- or down-regulated) between IR77298-14-1-2-B-10 and IR64 was not found; however, the expression patterns of a number of up-regulated genes (90 %) (e.g., SNAC subgroup) in the tolerant line were higher than in IR64 for all examined tissues (Supplementary Table 5). In addition, in the tolerant IR77298-14-1-2-B-10 line under severe drought stress, we noted that most of the genes (37 %) assigned to the SND subgroup were highly expressed in the root, and the SNAC (71 and 57 %) and NAM/CUC3 (72 and 27 %) genes were up-regulated in response to drought in the leaf and panicle when compared with normal growth conditions in Minghui 63 (Table 3). The results reported here suggest that up-regulation of the subgroup SND, SNAC and NAM/CUC3 genes may be involved in regulating the root, shoot, and panicle development and in the response to severe drought stress. However, further experiments are needed to evaluate these possibilities. To assess the accuracy of the microarray data, we selected 12 non-redundant DEGs and examined the similarity between gene responses observed by microarray and by RT-PCR (Fig. 9). Moreover, we confirmed this microarray data by qRT-PCR analysis on ten root tissue DEGs selected from different functional genes (Moumeni et al. 2011). Overall, the enrichment analysis suggests that different drought response strategies are used to achieve a drought response, as evidenced by this tolerant line. Therefore, it is vital to perform comparative analysis of gene expression profiles under both WDTs to determine the functional role of these genes in growth and response to stresses and to identify target genes involved in stress tolerance.

**Table 2:** Specific and overlapping up-regulated genes in tolerant line IR77298-14-1-2-B-10 (NIL10) compared with the sister line IR77298-14-1-2-B-13 (NIL13), control IR64, normal growth conditions (Minghui 63) and hormone treatments

NIL10-specific genes	NIL10	NIL13	IR64	Minghui 63	Hormone	Subgroup							
Root severe													
*Os07g04560*	1.726	–	–	–	–	ANAC34							
*Os10g38834*	1.223	–	–	–	–	SND							
Root mild													
*Os02g34970*	1.892	–	–	–	–	ONAC6							
*Os02g57650*	1.141	–	–	–	–	TIP							
*Os03g03540*	1.064	–	–	–	–	SND							
Leaf severe													
*Os01g28050*	1.075	–	–	–	–	ONAC2							
*Os01g29840*	1.18	–	–	12	–	NAM/CUC3							
Leaf mild													
*Os06g04090*	1	–	–	12	JA	SND							
*Os07g12340*	1.052	–	–	–	–	SNAC							
Panicle severe													
*Os03g12120*	1.069	–	–	–	–	ONAC4							
*Os03g59730*	1.623	–	–	16	–	ONAC3							
*Os06g15690*	1.081	–	–	–	–	ONAC4							
*Os08g06140*	1.16	–	–	–	–	TIP							
*Os08g33670*	1.614	–	–	–	–	NEO							
Panicle mild													
*Os01g70110*	1.361	–	–	–	–	ONAC1							
*Os04g43560*	1.234	–	–	–	SA	NAC22							
*Os08g10080*	1.948	–	–	16	–	NAC1							

Overlapped genes	Leaf severe			Leaf mild			Panicle severe			Panicle mild			Sub group
	NIL10	NIL13	IR64	NIL10	NIL13	IR64	NIL10	NIL13	IR64	NIL10	NIL13	IR64	
*Os05g34830*	3.72	3.74	3.48	2.35	2.24	1.86	1.09	1.96	1.77	1.89	1.6	1.09	SNAC
*Os11g03300*	2.84	1.99	2.3	3.45	2.85	2.73	4.56	3.66	3.66	4.84	4.25	2.97	SNAC
*Os12g03040*	2.72	1.754	2.165	3.29	2.81	2.59	4.21	3.40	3.38	4.69	4.28	2.72	SNAC

The vegetative and reproductive tissues of developmental stages under normal growth conditions are presented in “Materials and methods”. Different hormone stresses were administered at the seedling stage. None of the genes overlapped between root versus leaf and root versus panicle in all three genotypes, in Minghui 63, and under different hormone treatments

– Genes not differentially expressed or down-regulated in NIL13, IR64, Minghui 63, and hormone; *SA* salicylic acid; *JA* jasmonic acid

**Table 3:** The number of differentially expressed gene patterns in Minghui 63 under non-stress condition and severe stress conditions in drought-tolerant IR77298-14-1-2-B-10

Subfamily	No. of genes	Root		Leaf		Panicle	
		Up (%)	Down (%)	Up (%)	Down (%)	Up (%)	Down (%)
Minghui 63 under normal conditions							
TIP	10 (14)	2 (20)	0	1 (10)	0	0	0
**NAM/CUC3**	**13 (17)**	**3 (23)**	**0**	**2 (15)**	**1 (7)**	**0**	**1 (7)**
NAC1	5 (6)	1 (20)	0	1 (20)	0	1 (20)	0
NAC22	5 (5)	0	0	0	0	0	0
**SND**	**10 (14)**	**0**	**0**	**1 (10)**	**0**	**1 (10)**	**0**
ANAC34	10 (13)	0	1 (10)	1 (10)	0	0	1 (10)
**SNAC**	**14 (14)**	**1 (7)**	**1 (7)**	**0**	**2 (28)**	**0**	**2 (28)**
ONAC4	14 (14)	2 (28)	0	1 (7)	0	1 (7)	0
ONAC2	16 (16)	0	0	2 (12)	0	0	0
ONAC3	15 (17)	1 (6)	0	1 (6)	1 (6)	1 (6)	1 (6)
ONAC1	8 (8)	0	0	0	0	0	0
ONAC7	5 (5)	1 (20)	0	0	1 (20)	0	1 (20)
IR77298-14-1-2-B-10 under severe stress conditions							
TIP	9 (14)	2 (22)	3 (33)	2 (22)	1 (11)	4 (44)	1 (11)
**NAM/CUC3**	**11 (17)**	**0**	**3 (27)**	**8 (72)**	**1 (9)**	**3 (27)**	**3 (27)**
NAC1	4 (6)	0	2 (50)	2 (50)	1 (25)	0	3 (75)
NAC22	4 (5)	0	4 (100)	1 (25)	1 (25)	1 (25)	2 (50)
**SND**	**8 (14)**	**3 (37)**	**1 (12)**	**1 (12)**	**2 (25)**	**2 (25)**	**2 (25)**
ANAC34	9 (13)	1 (11)	5 (45)	0	7 (77)	2 (22)	0
**SNAC**	**14 (14)**	**1 (7)**	**5 (36)**	**10 (71)**	**0**	**8 (57)**	**2 (14)**
ONAC4	10 (14)	1(10)	1 (10)	2 (20)	0	**5 (50)**	3 (30)
ONAC2	10 (16)	2 (20)	1 (10)	3 (30)	3 (30)	0	2 (20)
ONAC3	11 (17)	0	2 (18)	1 (9)	1 (9)	2 (18)	3 (27)
ONAC1	4 (8)	0	3 (75)	1 (25)	3 (75)	1 (25)	1 (25)
ONAC7	4 (5)	1 (25)	3 (75)	1 (25)	3 (75)	1 (25)	1 (25)

Bold values indicate greater difference between normal and stressed conditions

() = the number of genes in each subfamily is indicated in parentheses, classification by Nuruzzaman et al. (2010). Selected tissues from Minghui 63, Root = seedling with 2 tillers, Leaf 1 = young panicle at stage 3, Panicle 1 = young panicle at stage 3, Up = up-regulated genes, Down = down-regulated genes

### Identification of the introgression regions in the IR64 background

Introgressed lines can be used as an important genetic material to decipher the physiological and molecular basis of tolerance in individual genomic regions. In different NILs, we found seven *OsNAC* genes introgressed (McNally et al. 2009) on chromosomes 1, 2, 7, 8, 10, and 11 in the IR64 background (Supplementary Fig. 1). From this genome-wide comparison, seven regions revealed differential hybridization; these regions were further genotyped with SSR markers to confirm polymorphism between the parental lines. We mapped four of the seven regions at 6.8–7.3, 6.7–7.2, 14.6–16.5 and 18.6–19.3 Mb on chromosomes 2, 4, 9 and 10, respectively, and these regions were finally selected for QTL identification (personal communication, Arvind Kumar). Of the seven genes, four (*Os01g23710*, *Os10g26270*, *Os08g2300*, and *Os11g31330*) were not differentially expressed under both WDTs, whereas two genes (*Os01g48130* and *Os07g27330*) were down-regulated in the panicle under 0.2 FTSW and in the root and panicle under 0.2FTSW and 0.5 FTSW. Only *Os02g57650* was up-regulated in the root under severe and mild stress conditions in both NILs (Supplementary Table 5). In these cases, a clear effect of the introgressed QTL of *OsNAC* genes on grain yield under drought conditions has not been confirmed. The *Os09g25070*, *Os09g24490*, and *Os06g48950* genes from WRKY, MYC, and ARF families were located in the QTL region (personal communication, Arvind Kumar). In the responses to abiotic stresses, four elements (BOXIINTPATPB, GT1CORE, IBOXCORE, and MYCATERD1) are linked to environmental stimuli (Fang et al. 2008), such as light and drought. We observed that *cis*-element motif of *OsNAC* genes harmonized with the above families and speculate that this factor may be involved in the drought response in tolerant NIL10. Further experiments using this NIL are ongoing to confirm this result. Marker-assisted mapping and introgression of major effect QTLs for grain yield under drought conditions may be a fast-track approach for developing drought tolerant varieties (Bernier et al. 2007), and several QTLs for yield and yield-related traits have been reported under drought stress on different rice chromosomes (Bernier et al. 2007; Venuprasad et al. 2009).

### Consensus *cis*-regulatory elements in both NILs

The *cis*-regulatory DNA sequences control gene responses in different tissues and constitute the essential functional linkage between gene regulatory networks. We performed *cis*-element analysis in the promoter sequences (2 kb upstream region) of specific DEGs in the tolerant line using Multiple Em for Motif Elicitation (MEME) and a rice *cis*-element searching tool (RiCES; Doi et al. 2008). We determined that *cis*-motifs matching to GT-1, BPBF, WRKY, and MYC in NIL10 and ARF in NIL18 were the most abundant *cis*-elements under both WDTs. In the responses to abiotic stresses, four elements (BOXIINTPATPB, GT1CORE, IBOXCORE, and MYCATERD1) were linked to environmental stimuli, such as light and drought. Most of the *OsNAC* genes with tissue-specific expression profiles contained at least three of these *cis*-elements (Supplementary Table 8), whereas uncommon *cis*-elements were found in the genes in the drought-susceptible line (data not shown). With the help of bioinformational analysis, we predict that further analysis of a number of the above transcription factors will contribute to a deeper understanding of gene regulation in rice under different drought stress conditions.

## Discussion

Overall, we performed the global expression patterns of members of the *OsNAC* gene family under normal growth conditions, different hormones, and severe and mild drought stress conditions in different rice genotypes. Most of the members belonging to the SNAC, NAM/CUC3, and SND subgroups were activated in the specific tissue(s) under drought stress conditions in the tolerant line. These results provide a new avenue to select the best candidate gene for further function analysis.

### Diverse expression of the *OsNAC* gene family

The objectives of this study were to determine the expression patterns of members of the *OsNAC* gene family under two drought stress conditions between the two NILs. We compared the genes responding to normal growth conditions or two drought stress conditions (investigation of gene responses in the drought tolerant line in specific tissues is a novel contribution) to identify the most promising genes or subgroups of genes based on their expression profiles in specific tissues under normal or different stress conditions. We also aimed to identify the drought response *cis*-element, which may assist the understanding of the function of these novel genes in network systems, which in turn may help to understand the function of these novel genes in biological processes or in gene network systems. In conclusion, experimental support of a casual relationship between possible candidate *NAC* gene expression patterns and drought tolerance is of fundamental and practical interest for understanding the genetic control of this complex trait. The number of tissues and treatments used in this study may have contributed to the identification of stably expressed genes. Analyses of members of this gene family have revealed a wealth of features and phenomena regarding their gene expression, with fundamental implications for the biological processes underlying growth and development. The wide expression patterns of a number of OsNAC members suggest that these genes may play regulatory roles at multiple development stages, whereas the unique expression patterns of other members indicate that these genes participate in specific biological processes. For example, *Os07g37920* and *Os01g01430* were expressed preferentially in the leaf and panicle; therefore, it is possible that these genes are involved in the regulation of leaf and panicle growth (Fig. 1a, Supplementary Table 2). *Os11g03300*/*OsNAC10* is highly expressed predominantly in the root and leaf (Jeong et al. 2010). In transgenic plants under normal growth conditions, the temporal and spatial expression patterns of *Os03g60080/SNAC1* has been investigated in the callus, root, ligule, stamen, and pistil tissues, and it is predominantly expressed in guard cells in rice under drought conditions (Hu et al. 2006). *Os03g56580* demonstrated preferential expression in the stamen 1 day before flowering suggesting important roles in pollination and fertilization (Fig. 1a, Supplementary Table 2). However, the number of genes expressed in the stamen is relatively small, and these genes have a unique transcriptional profile compared with other tissues. In Arabidopsis, pollen has a unique expression profile compared with other vegetative and generative organs (Becker et al. 2003). Thus, pollen is the major site of variations in the expression levels for many genes (Czechowski et al. 2005). The preferential expression of three genes, *Os01g01430*, *Os02g38130*, and *Os12g22940* in the panicle, *Os03g56580* in the stamen and two genes, *Os05g34310* and *Os02g18460,* in the endosperm, suggests that these genes might be involved in panicle or endosperm development (Fig. 1a, Supplementary Table 2). Hu et al. (2006) revealed that *Os03g60080/SNAC1* promotes stomata closure in the flag leaf when overexpressed under drought stress. Therefore, these genes, which are preferentially expressed in specific tissues, may deserve special attention in further functional investigations. Overall, *OsNAC* gene expression was highest in the leaf 2 and lowest in the endosperm 3 (Fig. 1b). Generating global gene expression profiling data covering different tissues and genotypes promotes the understanding of gene functions in the gene network pathways.

### Responses of *OsNAC* genes to various treatment conditions

Abiotic stresses trigger a wide range of plant responses, from the alteration of gene expression and cellular metabolism to changes in plant growth and development, and crop yields. The expression of members of the *OsNAC* gene family under hormone treatment requires extensive cross-talk between the response pathways, and it is likely that substantial physiological connections exist between NAC protein production and phytohormone treatments. A high expression profile of a specific gene under these conditions suggests that the genes play an important role in protection against stressful hormone treatments. A number of *NAC* genes (e.g., *AtNAC2*) in plants are affected by auxin, ethylene (Xie et al. 2000; He et al. 2005), and ABA (e.g., *OsNAC5*; Sperotto et al. 2009). The expression of different types of proteins increases or decreases following GA3 treatment in rice leaf sheath (Shen et al. 2002). In Arabidopsis, NAC TF *NTL8* regulates GA3-mediated salt signaling in seed germination (Kim et al. 2008). ABA plays a major role in mediating the adaptation of the plant to stress, and this hormone can stimulate root growth in plants that need to increase their ability to extract water from the soil. In this study, *Os05g34830* (SNAC) was induced specifically in the root in the tolerant NIL10 under both drought conditions and was induced in the leaf and panicle under both WDTs in both NILs. *Os05g34830* is also activated by ABA treatment (Supplementary Tables 4 and 7). Therefore, this gene might be involved in water uptake under dehydrated soil conditions. *OsNAC5*/*ONAC009*/*ONAC071* and *OsNAC6* are homologs that are induced by abiotic stresses, such as drought, high salinity, and ABA (Takasaki et al. 2010). *AtNAC1* and *AtNAC2* are induced by auxin and ABA, respectively, and *AtNAC1* mediates auxin signaling to promote lateral root development in Arabidopsis (Xie et al. 2000; He et al. 2005). ABA signaling induces the expression of genes encoding proteins that protect cells in the vegetative tissues from damage when they become dehydrated. Nuclear protein X1 (NPX1) is up-regulated by stress and treatment with exogenous ABA in Arabidopsis, and it is involved in stomatal closure, seed germination, and primary root growth. These well-known ABA responses are less sensitive to ABA in NPX1-overexpressing plants (Kim et al. 2009). In this study, *Os03g21030*, *Os05g34830*, and *Os07g48550* were induced in the root, leaf, and panicle under both drought conditions and ABA treatments. The higher levels of ABA found in water-stressed leaves, which activates potassium ions to be transported out of the guard cells, causes the stomata to close, and water is reserved in the leaf when soil water is deficient. From these results, we speculate that *OsNAC* genes function in ABA signaling pathways and in the defensive response against water deficit. *Os03g60080*/*SNAC1* promoted stomatal closure under drought stress (Hu et al. 2006). We observed that *Os03g60080*/*SNAC1* was up-regulated following treatment with GA3 and SA in both lines under both WDTs in the leaf and panicle, and the expression intensity of the gene was much greater in the tolerant line. Similarly, in the leaf and panicle, *Os01g66120*/*SNAC2/6* was common to both NILs and was up-regulated by NAA, GA3 and KT; however, the expression intensity of this gene was much greater in NIL10 than in NIL13 under both WDTs. In Arabidopsis, the expression of the *RD26* gene is induced not only by drought but also by ABA and high salinity (Fujita et al. 2004). Although it is not possible to configure such a complex regulatory network based only on the expression patterns obtained in this study, understanding the changes in the regulatory elements may be important for understanding the responses to different hormone treatments. In this study, several *OsNAC* genes exhibited high or low expression under different hormone treatments in the seedlings (Fig. 3, Supplementary Tables 3 and 4).

### *OsNAC* gene expression profiles under 0.2 FTSW and 0.5 FTSW in NILs

This study focused on the identification and expression profiles of drought stress-responsive genes from rice genotypes at different developmental stages under different stress conditions. In the whole rice genome, approximately 7 % of the genes are induced by drought or salt stresses (Zhou et al. 2007), whereas in the *OsNAC* family, at least 68 % of the genes were induced by both WDTs. In this respect, we investigated the DEGs in the tolerant line compared with its sister susceptible line and IR64 and focused more on the genes exclusively up-regulated in NIL10 compared with NIL13. Therefore, despite the common genetic background of the rice NILs as backcross progeny from Aday Sel. × IR64, which is approximately 98 and 97 % for IR77298-5-6-B and IR77298-14-1-2-B families, respectively (Venuprasad et al. 2011), the tolerant NIL10 exhibited distinctive differences in its GEPs in response to drought. To explore the question of overlap in stress-responsive genes in more detail, we specifically investigated how many genes were differentially up-regulated under all stress conditions and how many genes responded significantly to a single stress (Fig. 3, Supplementary Tables 3–7). Under the both stress conditions, we determined that a greater number of DEGs, unique up-regulated genes, and a much greater intensity of gene expression was observed in the tolerant line compared with the susceptible line or IR64 in different tissues (Figs. 5, 6, Supplementary Table 7). The expression of unique genes or the increased expression intensity of genes is thought to be related to the drought response in a particular tissue. Kubo et al. (2005) first discovered the NAC domain TFs, VASCULAR-RELATED NAC-DOMAIN7 (VND7) and VND6, as “master activators” of secondary cell wall formation in proto- and metaxylem vessels, respectively. The NAC family proteins SECONDARY WALL-ASSOCIATED NAC DOMAIN1 (SND1) and NAC SECONDARY WALL THICKENING PROMOTING FACTOR1 (NST1) redundantly activate Arabidopsis fiber secondary cell wall formation (Mitsuda et al. 2007; Zhong et al. 2007). Bennett et al. (2010) presented a striking example of NAC TFs regulating primary cell wall modification in the root cap. In our microarray results, we identified two genes (*Os10g38834* and *Os03g03540*, members of the SND subgroup) that were differentially up-regulated in the root in the tolerant line (Table 2). We determined that nine genes (e.g., *Os01g01430* and *Os01g70110*) were highly activated in the panicle in NIL10, whereas six genes (e.g., *Os02g12310* and Os02g57650), assigned to the SNAC and TIP subgroups, were up-regulated with high levels of expression in the root and leaf under severe stress in IR77298-14-1-2-B-10 compared with IR77298-14-1-2-B-13 or IR64 (Fig. 6a, b). Similar conclusions have been drawn from analyses of promoter-GUS fusions of the cold-inducible *Os01g66120/SNAC2/6*, *Os11g03300/OsNAC10*, *RD29A*, *COR15A*, *KIN1*, and *COR6.6* genes in rice and Arabidopsis, which are regulated during plant development (root, leaf, and pollen) under both stressed (drought and cold) and unstressed conditions (Hu et al. 2008; Jeong et al. 2010). Lan et al. (2005) determined that a large portion of the genes regulated by dehydration are also up-regulated by pollination/fertilization. In transgenic rice, *Os01g66120/OsNAC2/6* and *Os11g03300/OsNAC10* genes enhanced drought and salt tolerance (Nakashima et al. 2009; Jeong et al. 2010), and *Os03g60080/SNAC1* increased grain yield (21–34 %) under drought stress (Hu et al. 2006). A number of genes belonging to the SND, SNAC and NAM/CUC3 subgroups were up-regulated in the root, leaf and panicle under severe stress in NIL10 compared with Minghui 63 (Table 3). Udupa et al. (1999) reported that comparative gene expression profiling is an efficient way to identify pathways and genes regulating a stress response under different stress conditions. The Arabidopsis *NAC* gene *ANAC092* demonstrates an intricate overlap of *ANAC092*-mediated gene regulatory networks during salt-promoted senescence and seed maturation (Balazadeh et al. 2010). In our study, a number of novel genes were chosen for their higher expression in a particular tissue and their more specific expression in tolerant line (Figs. 5, 6; Table 2). Taken together, these results add to our knowledge of the involvement of *OsNAC* genes in plant responses to drought and show that members of certain subgroups or members of the *OsNAC* gene family might be involved in protection against drought stress, although further research will be required to validate this hypothesis.

### Defense mechanism of *OsNAC* genes in rice

Transcription factors and *cis*-elements function in the promoter region of different stress-related genes, and overexpression of these genes may improve the plant’s tolerance to stress. We predicted that a number of gene-specific *cis*-elements might be important in the regulation of target genes by other factors and that these *cis*-elements may influence the introgressed regions of the drought QTLs. In response to drought, the NAC TF regulates many target genes by binding to the CATGTG motif in the promoter region of the candidate gene and activating transcription (Nakashima et al. 2007); this transcriptional regulatory system is known as a regulon. ABA is produced under drought stress conditions and plays a crucial role in drought tolerance in plants (Shinozaki et al. 2003). A single TF can control the expression of many target genes by binding to the *cis*-elements in the promoters (Fig. 7). Including NAC and other regulons, *OsDREB2* responds to dehydration in rice (Dubouzet et al. 2003), dehydration-responsive element binding protein 1 (DREB1)/C-repeat binding factor (CBF) DREB2 regulons function in ABA-independent gene expression, whereas the ABA-responsive element (ABRE) binding protein (AREB)/ABRE binding factor (ABF) regulon functions in ABA-dependent gene expression. ABA-activated OSRK1 protein kinases phosphorylate and activate the AREB/ABF-type proteins in rice (Chae et al. 2007). Both ABA-independent and ABA-dependent signal transduction pathways convert the initial stress signal into cellular responses. The members of TF families that are involved in both ABA-independent (AP2/ERF, bHLH, and NAC) and ABA-dependent (MYB, bZIP, and MYC) pathways are up-regulated in rice. TFs belonging to this family of genes interact with specific *cis*-elements and/or proteins, and their overexpression confers stress tolerance in heterologous systems (Fujita et al. 2004; Tran et al. 2004; Hu et al. 2006). In this study, we found that several *OsNAC* genes (e.g., *Os05g34830* and *Os07g48550*) involved in the ABA treatment were members of the SNAC and NAM/CUC3 subgroups and contained *cis*-elements (ABRE, ACGTGGTC and ACGTGKC) in their upstream regions. Moreover, their expression may be induced by stress (Fig. 7). Microarray analyses have revealed that up-regulated genes in rice plants overexpressing *Os11g03300*/*OsNAC10* were drought stress tolerant at the reproductive stage and demonstrated increased grain yield (25–42 % more than controls) in the field under drought and normal conditions (Jeong et al. 2010). Expression of *OsNAC6* is induced by ABA and abiotic stresses, including cold, drought, and high salinity (Nakashima et al. 2007). It was shown that stress-inducible promoters, such as the *OsNAC6* and *SNAC1* promoters, are more suitable for overexpression to minimize negative effects on plant growth in transgenic rice. The *ONAC010* gene encodes a protein that demonstrates high homology to the NAC protein NAM-B1, which regulates senescence, and was shown to improve grain protein, zinc, and iron content in wheat (Uauy et al. 2006). In our study, the regulation of *OsNAC* genes was very precise in terms of the spatial and temporal distribution. Thus, comparative analysis of gene expression profiles under both WDTs to determine the functional role of *OsNAC* genes in the growth of the plant and its response to stress as well as the identification of target genes for TFs involved in stress responses is important.

The application of a new comprehensive 44K oligoarray platform with different rice genotypes enabled us to determine gene expression patterns in different tissues of two NILs with contrasting yield performances under drought stress at the reproductive stage. By comparing the gene expression profiles of all genotypes under drought stress and unstressed conditions, we identified several putative genes in a specific tissue that may be responsible for the drought response in the drought-tolerant line. These genes should be considered novel reference genes, and a number of putative *cis*-elements were indentified, which may help to understand the function of these key genes in network pathways. Together, these data provide a useful reference and establish a starting point for determining the functions of the *OsNAC* family of genes in rice at the reproductive stage of growth. The genes belonging to the SNAC and NAM/CUC3 subgroups were activated in the leaf and panicle tissues, and the SND subgroup genes were involved in the root. Overexpression and knockdown/mutant analyses of particular members of this gene family are underway in our laboratory to investigate optimal molecular breeding schemes for the *OsNAC* gene family.

## Electronic supplementary material

Below is the link to the electronic supplementary material.

Supplementary material 1 (DOC 36 kb)

Supplementary material 2 (XLS 216 kb)

Supplementary material 3 (XLS 58 kb)

Supplementary material 4 (XLS 28 kb)

Supplementary material 5 (XLS 49 kb)

Supplementary material 6 (PPT 378 kb)

Supplementary material 7 (XLS 58 kb)

Supplementary material 8 (DOC 48 kb)

Supplementary material 9 (PPT 300 kb)
