# Supplementary material for: Comprehensive gene expression analysis of the NAC gene family under normal growth conditions, hormone treatment, and drought stress conditions in rice using near-isogenic lines (NILs) generated from crossing Aday Selection (drought tolerant) and IR64
Source: Mol Genet Genomics. 2012 Apr 12;287(5):389–410. doi: 10.1007/s00438-012-0686-8 (PMC3336058; doi:10.1007/s00438-012-0686-8)
Supplement: Supplementary file 1 — Supplementary material 1 (DOC 36 kb) [file 438_2012_686_MOESM1_ESM.doc]

Supplemental Table 1 Gene-specific primers used for RT-PCR analysis

| Gene name | Forward primer (5'-------------3') | Reverse primer (5'-------------3') |
| --- | --- | --- |
| *Os01g66120* | CAATGTTGACGGGTCAATGA | TCGCCGCCAGTCCTGGACGT |
| *Os01g70110* | AGCTCCTGCTCCACTACCTC | GCGTGGTGGTCTTCTTGAAT |
| *Os01g01430* | CAGCAGCTGCAGCACTACAT | GGATGTGTCGTCGTTGTGAG |
| *Os02g12310* | CGACTACTTGCAGAGGCGTA | GGAAGACGATTCACCGAAGA |
| *Os02g18460* | TACAGCGAACAAAGCCAGTG | TCGCAGTACGCGTATTCTTG |
| *Os02g57650* | GGAGGAAGATTGGGAAGAGG | GGGTCAATCCAATTCACAGG |
| *Os02g34970* | ATGAAGAGCTCGTCGTCCAT | TGCTTGAGTGGCCTCTCTTC |
| *Os02g15340* | AGGATGGGTGGTGTGTAGG | AGAGCTGGTCGAGGTTCTG |
| *Os03g60080* | GCCGAGGTGGATCTCTACAA | GTTGTCCACGATCTCCGACT |
| *Os04g35660* | GTTTCCACTTCTTCCCGTCA | GCTTGCTTGAACTGCTTGAA |
| *Os04g39960* | GATGAAGGAGTACCGGCTCA | GCTCATCCTCGTCGGAGTAG |
| *Os05g34310* | CCACCCATCCTTGTCTTCAT | GCTGGTGTCCTCTTCCTCAA |
| *Os06g04090* | TCTTCCCCAATGCACGGT | GGTCGTCAAGTCTGCGTGTAG |
| *Os08g10080* | GACGTCGACCTCAACAAGTG | AGATGATGGGCTTGGAATTG |
| *Actin* | CAATGAGCTTCGTGTTGCAC | GGCACCTGAACCTTTCTGAC |
