# Supplementary material for: Comprehensive gene expression analysis of the NAC gene family under normal growth conditions, hormone treatment, and drought stress conditions in rice using near-isogenic lines (NILs) generated from crossing Aday Selection (drought tolerant) and IR64
Source: Mol Genet Genomics. 2012 Apr 12;287(5):389–410. doi: 10.1007/s00438-012-0686-8 (PMC3336058; doi:10.1007/s00438-012-0686-8)
Supplement: Supplementary file 6 — Supplementary material 6 (PPT 378 kb) [file 438_2012_686_MOESM6_ESM.ppt]

## Slide 1
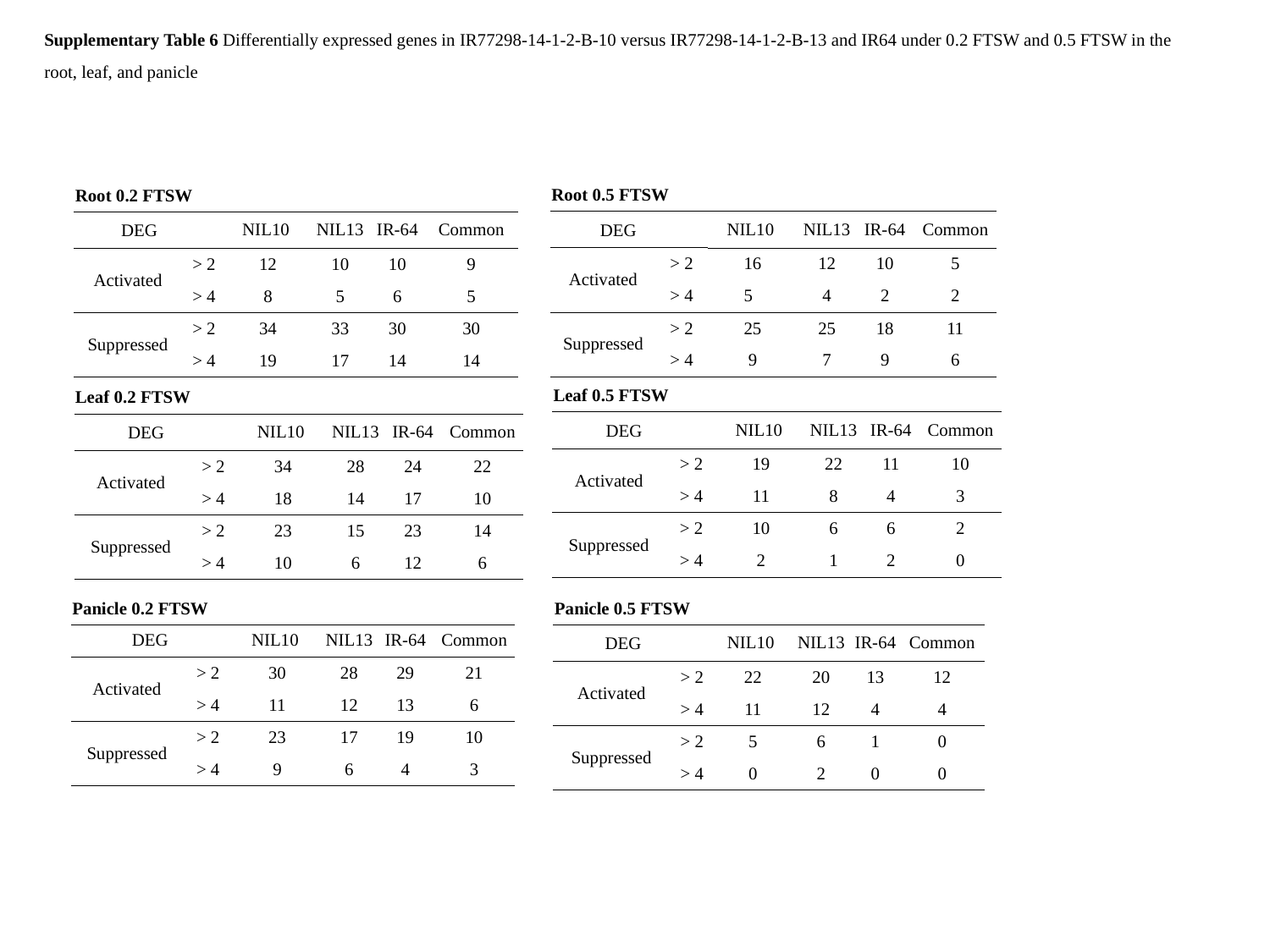

Supplementary Table 6 Differentially expressed genes in IR77298-14-1-2-B-10 versus IR77298-14-1-2-B-13 and IR64 under 0.2 FTSW and 0.5 FTSW in the root, leaf, and panicle
| Root 0.5 FTSW | | | | | |
| --- | --- | --- | --- | --- | --- |
| DEG | | NIL10 | NIL13 | IR-64 | Common |
| Activated | > 2 | 16 | 12 | 10 | 5 |
| | > 4 | 5 | 4 | 2 | 2 |
| Suppressed | > 2 | 25 | 25 | 18 | 11 |
| | > 4 | 9 | 7 | 9 | 6 |
| Root 0.2 FTSW | | | | | |
| --- | --- | --- | --- | --- | --- |
| DEG | | NIL10 | NIL13 | IR-64 | Common |
| Activated | > 2 | 12 | 10 | 10 | 9 |
| | > 4 | 8 | 5 | 6 | 5 |
| Suppressed | > 2 | 34 | 33 | 30 | 30 |
| | > 4 | 19 | 17 | 14 | 14 |
| Leaf 0.5 FTSW | | | | | |
| --- | --- | --- | --- | --- | --- |
| DEG | | NIL10 | NIL13 | IR-64 | Common |
| Activated | > 2 | 19 | 22 | 11 | 10 |
| | > 4 | 11 | 8 | 4 | 3 |
| Suppressed | > 2 | 10 | 6 | 6 | 2 |
| | > 4 | 2 | 1 | 2 | 0 |
| Leaf 0.2 FTSW | | | | | |
| --- | --- | --- | --- | --- | --- |
| DEG | | NIL10 | NIL13 | IR-64 | Common |
| Activated | > 2 | 34 | 28 | 24 | 22 |
| | > 4 | 18 | 14 | 17 | 10 |
| Suppressed | > 2 | 23 | 15 | 23 | 14 |
| | > 4 | 10 | 6 | 12 | 6 |
| Panicle 0.2 FTSW | | | | | |
| --- | --- | --- | --- | --- | --- |
| DEG | | NIL10 | NIL13 | IR-64 | Common |
| Activated | > 2 | 30 | 28 | 29 | 21 |
| | > 4 | 11 | 12 | 13 | 6 |
| Suppressed | > 2 | 23 | 17 | 19 | 10 |
| | > 4 | 9 | 6 | 4 | 3 |
| Panicle 0.5 FTSW | | | | | |
| --- | --- | --- | --- | --- | --- |
| DEG | | NIL10 | NIL13 | IR-64 | Common |
| Activated | > 2 | 22 | 20 | 13 | 12 |
| | > 4 | 11 | 12 | 4 | 4 |
| Suppressed | > 2 | 5 | 6 | 1 | 0 |
| | > 4 | 0 | 2 | 0 | 0 |
