# Supplementary material for: Comprehensive gene expression analysis of the NAC gene family under normal growth conditions, hormone treatment, and drought stress conditions in rice using near-isogenic lines (NILs) generated from crossing Aday Selection (drought tolerant) and IR64
Source: Mol Genet Genomics. 2012 Apr 12;287(5):389–410. doi: 10.1007/s00438-012-0686-8 (PMC3336058; doi:10.1007/s00438-012-0686-8)
Supplement: Supplementary file 8 — Supplementary material 8 (DOC 48 kb) [file 438_2012_686_MOESM8_ESM.doc]

Supplementary Table 8 Putative *cis*-elements enriched in promoters of rice *NAC* genes

| *cis*-element name | Sequence | TF | Stimulus/tissue |
| --- | --- | --- | --- |
| GAGAGMGSA1 | GAGAGAGAGAGAGAGAGA |  |  |
| GAGA8HVBKN3 | GAGAGAGAGAGAGAGA |  |  |
| RYREPEATGMGY2 | CATGCAT |  |  |
| RYREPEATLEGUMINBOX | CATGCAY |  | Seed |
| BOXIINTPATPB | ATAGAA | GT-1 | Light |
| RYREPEATVFLEB4 | ACGTGGTC | ABRE | ABA, drought |
| SV40COREENHAN | GTGGWWHG |  |  |
| ARFAT | TGTCTC | ARF | Auxin |
| PYRIMIDINEBOXHVEPB1 | TTTTTTCC |  | GA3 |
| TATABOX4 | TATATAA |  |  |
| POLASIG2 | AATTAAA |  |  |
| GT1CORE | GGTTAA | GT-1 | Light |
| RYREPEATBNNAPA | CATGCA |  | Seed |
| PYRIMIDINEBOXOSRAMY1A | CCTTTT | BPBF | GA3 |
| IBOXCORE | GATAA |  | Light |
| WBBOXPCWRKY1 | TTTGACY | WRKY | SA |
| LTRE1HVBLT49 | CCGAAA |  | Cold |
| [G-box-like](http://www.bioinformatics2.wsu.edu/Osiris/binding_factors/Gboxlike.html) | CACGTG |  |  |
| [POLASIG1](http://www.bioinformatics2.wsu.edu/Osiris/binding_factors/POLASIG1.html) | AATAAA |  |  |
| [TATABOXOSPAL](http://www.bioinformatics2.wsu.edu/Osiris/binding_factors/TATABOXOSPAL.html) | CACATG |  |  |
| [TATAboxII](http://www.bioinformatics2.wsu.edu/Osiris/binding_factors/TATAboxII.html) | TATTAATA |  |  |
| MYBGAHV | TAACAAA | MYB | GA |
| MYB1AT | WAACCA |  | Drought |
| [MYCATERD1](http://www.bioinformatics2.wsu.edu/Osiris/binding_factors/MYCATERD1.html) | CATGTG | MYC | Drought |
| MYCATRD22 | CACATG | MYC | ABA, drought |
| TATCCAYMOTIFOSRAMY3D | TATCCAY |  |  |
| ACGTABREMOTIFA2OSEM | ACGTGKC | ABRE | ABA, seed |
| BRRE | GTG TCG |  |  |
| PBF | WAAAGNG |  |  |
| SURE | GAGAC |  |  |
| ABREOSRAB21 | ACGTSSSC | ABRE | ABA |
