# Supplementary material for: Comprehensive gene expression analysis of the NAC gene family under normal growth conditions, hormone treatment, and drought stress conditions in rice using near-isogenic lines (NILs) generated from crossing Aday Selection (drought tolerant) and IR64
Source: Mol Genet Genomics. 2012 Apr 12;287(5):389–410. doi: 10.1007/s00438-012-0686-8 (PMC3336058; doi:10.1007/s00438-012-0686-8)
Supplement: Supplementary file 9 — Supplementary material 9 (PPT 300 kb) [file 438_2012_686_MOESM9_ESM.ppt]

## Slide 1
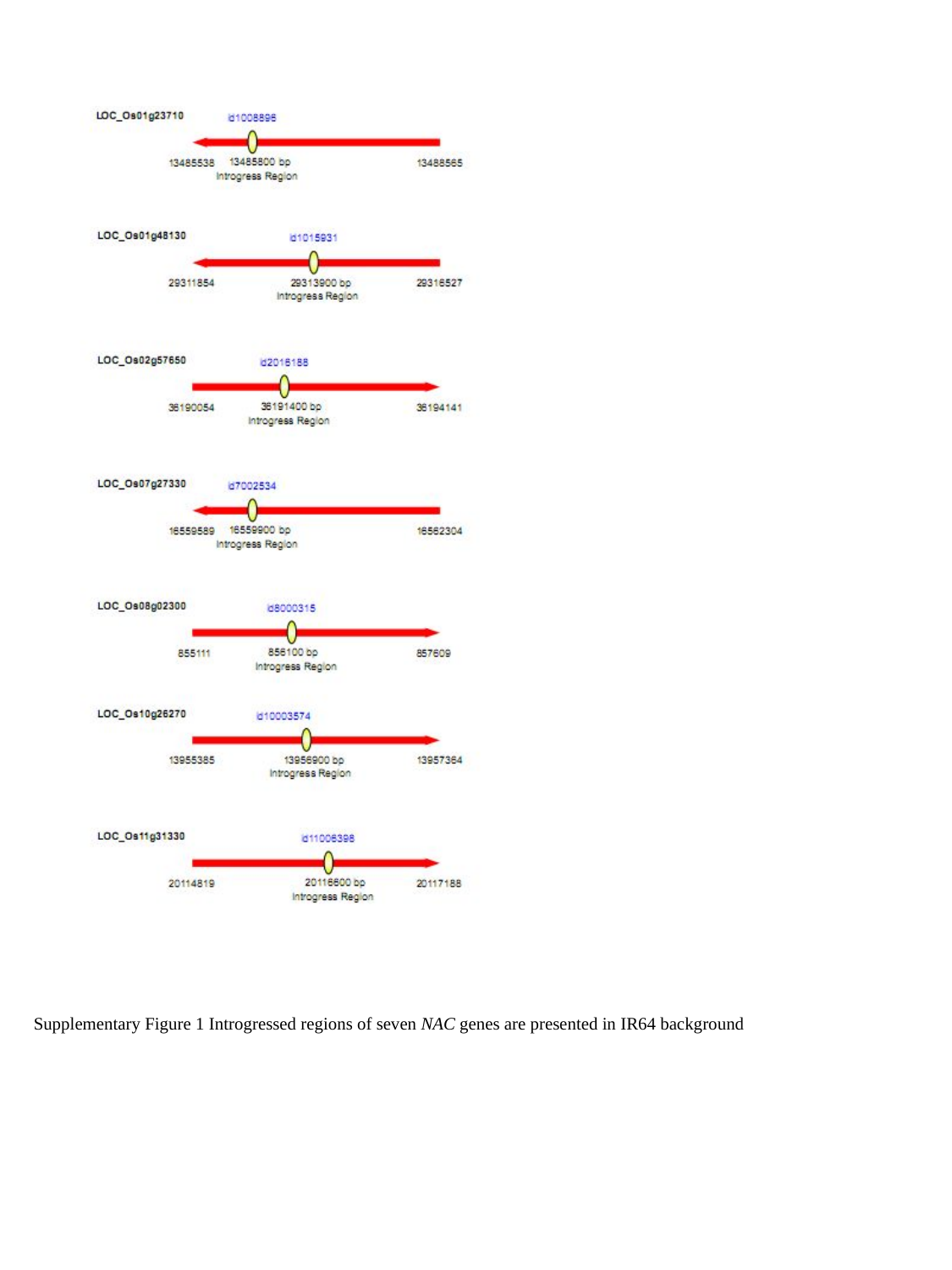

Supplementary Figure 1 Introgressed regions of seven NAC genes are presented in IR64 background
